# The benefits, costs and feasibility of a low incidence COVID-19 strategy

**DOI:** 10.1016/j.lanepe.2021.100294

**Published:** 2022-01-02

**Authors:** Thomas Czypionka, Emil N. Iftekhar, Barbara Prainsack, Viola Priesemann, Simon Bauer, André Calero Valdez, Sarah Cuschieri, Enrico Glaab, Eva Grill, Jenny Krutzinna, Christos Lionis, Helena Machado, Carlos Martins, George N. Pavlakis, Matjaž Perc, Elena Petelos, Martyn Pickersgill, Alexander Skupin, Eva Schernhammer, Ewa Szczurek, Sotirios Tsiodras, Peter Willeit, Paul Wilmes

**Affiliations:** a Institute for Advanced Studies, Vienna, Austria, and London School of Economics and Political Science, London, UK; b Max Planck Institute for Dynamics and Self-Organization, Göttingen, Germany; c University of Vienna, Vienna, Austria; d Max Planck Institute for Dynamics and Self-Organization, Göttingen, Germany; e Max Planck Institute for Dynamics and Self-Organization, Göttingen, Germany; f RWTH Aachen University, Aachen, Germany; g Faculty of Medicine and Surgery, University of Malta, Msida, Malta; h University of Luxembourg, Esch-sur-Alzette, Luxembourg; i Ludwig-Maximilians University, Munich, Germany; j University of Bergen, Bergen, Norway; k Clinic of Social and Family Medicine, Faculty of Medicine, University of Crete, Heraklion, Greece and Institute of Health and Medicine, University of Linkoping, Linkoping, Sweden; l University of Minho, Minho, Portugal; m Department of Community Medicine, Health Information and Decision Sciences of the Faculty of Medicine of the University of Porto, Porto, Portugal; n National Cancer Institute, Bethesda, USA; o University of Maribor, Maribor, Slovenia, and Department of Medical Research, China Medical University Hospital, China Medical University, Taichung, Taiwan; p Clinic of Social and Family Medicine, Faculty of Medicine, University of Crete, Heraklion, Greece and Faculty of Health, Medicine and Life Sciences, Maastricht University, Maastricht, The Netherlands; q The University of Edinburgh, Edinburgh, UK; r University of Luxembourg, Esch-sur-Alzette, Luxembourg; s Medical University of Vienna, Vienna, Austria; t University of Warsaw, Warsaw, Poland; u National and Kapodistrian University of Athens Medical School, Athens, Greece; v Medical University of Innsbruck, Innsbruck, Austria, and University of Cambridge, Cambridge, UK; w University of Luxembourg, Esch-sur-Alzette, Luxembourg

## Abstract

In the summer of 2021, European governments removed most NPIs after experiencing prolonged second and third waves of the COVID-19 pandemic. Most countries failed to achieve immunization rates high enough to avoid resurgence of the virus. Public health strategies for autumn and winter 2021 have ranged from countries aiming at low incidence by re-introducing NPIs to accepting high incidence levels. However, such high incidence strategies almost certainly lead to the very consequences that they seek to avoid: restrictions that harm people and economies. At high incidence, the important pandemic containment measure ‘test-trace-isolate-support’ becomes inefficient. At that point, the spread of SARS-CoV-2 and its numerous harmful consequences can likely only be controlled through restrictions. We argue that all European countries need to pursue a low incidence strategy in a coordinated manner. Such an endeavour can only be successful if it is built on open communication and trust.

## Introduction

In light of decreasing COVID-19 infection rates in early summer 2021, governments in many European countries removed most non-pharmaceutical interventions (NPIs) aimed at controlling the pandemic. In addition to the growing (deceptive) sense of complete safety that the progress in vaccination programmes conveys, there is also considerable pressure on policymakers to “give people back their freedom”. This pressure is rising amidst growing frustration about the protracted pandemic and loss of public trust in governments.^1^^,^^2^

While the rate of fully vaccinated people is not sufficient to interrupt infection chains and reduce infection rates in most European countries, especially in the East and among young people, and emerging variants of concern (VOCs) show partial immune escape, lifting certain NPIs means living with a relatively high incidence of cases. Such high incidence means hundreds of cases per week per 100,000 people. While in some countries case numbers have begun to drop, the first 18 months of the pandemic have taught us that it is extremely difficult, if not impossible, to maintain stable incidence at intermediate levels, especially given the increased reproduction number of the Delta variant. Pursuing a low-incidence strategy consequently seems warranted for the winter, especially when people spend more time indoors. *However, achieving a low COVID-19 incidence across Europe will continue to be impossible without good communication, public trust, and a coordinated pan-European approach*.^3^,^30^

## Search strategy and selection criteria

Work for this study has emerged from a previous Delphi study led by Viola Priesemann and Emil Iftekhar.^4^ The scientific procedure of the Delphi study consisted of bias-avoiding discussions between scientists of various disciplines and European countries. These discussions were guided, summarised, and synthesized by facilitators. For the insights of this work, the participants of said Delphi study analysed different public health policy strategies against the backdrop of the advantages and disadvantages of different possible scenarios developed in the Delphi study. The considered content arose from three iterations of the authors providing input and evidence and subsequent evaluation by the main authors.

## Hastily reducing all NPIs means accepting high COVID-19 incidence

NPIs encompass a wide range of measures and policies, from practices with little impact on personal freedom (e.g., regular disinfection of public spaces) to those that many people consider highly restrictive or invasive (e.g., restrictions on movement). In many countries politicians felt compelled to abolish mask mandates the moment infection numbers declined.

Examples from Israel and Singapore suggest, however, that even in countries with high vaccination rates, especially when colliding with waning immunity, lifting all NPIs will contribute to high incidence and associated undesirable effects.^5^, ^6^, ^7^, ^8^ Other factors contributing to rising incidence in the winter months include travel and cross-border commutes in and out of regions with high incidence; disadvantageous seasonality effects^9^, ^10^, ^11^; insufficient support for people to enable self-isolation; lowered risk perception, and inadequate governmental communication around harm reduction^10^^,^^11^; and the emergence of VOCs with partial immune escape, such as Delta.^6^^,^^12^^,^^13^

Against this backdrop, repealing most NPIs appears to be a risky strategy. At incidence levels exceeding 50 cases per 100,000 people per week, test-trace-isolate-support systems (TTIS) capacity is quickly exceeded. This makes it impossible to detect and break many chains of infection. A further rapid increase in incidence to the point of complete loss of control over transmission can then potentially result.^12^^,^^13^ Exempting vaccinated people from NPIs, such as mask wearing or testing, poses further challenges to containment. This is because these individuals may still get infected and transmit the virus; given frequent exemption of testing requirements for vaccinated people on the basis of European Union's Digital Covid Certificate, their role in transmission chains needs to be assessed in terms of contribution to the spread of VOCs, particularly given Delta's higher transmissibility. Without effective TTIS systems, infections will rise unreported, and many infection chains will not be detected and broken in time. If this happens in winter 2021/2022, incidence could reach levels as high as 500 cases per week per 100,000 people.^16^

## The costs of high incidence

The first 18 months of the pandemic have taught us that accepting a high incidence of COVID-19 is unwise - even since vaccines have been available. First, a high incidence incurs direct harm to the health of considerable parts of the population - particularly the most vulnerable, including economically deprived and/or socially marginalised populations,^17^ who tend to be less well-served by vaccination programmes and campaigns.^18^ The harms include a higher COVID-19 associated mortality and more cases of Long COVID, including pulmonary, cardiovascular, and renal sequelae.^19^^,^^20^ Many people either cannot be vaccinated for health reasons or show poor immune response to the vaccine (e.g., people with weakened immune systems), and thus remain at risk.^21^

Second, a high incidence also has negative impacts on the workforce; when people fall ill or need to isolate or quarantine, others need to do their work. This additional workload increases the likelihood of burnout, as has become evident especially among healthcare workers.^22^ Assuming an incidence of 100 new infected persons per 100,000 per week among the working population, and a quarantine period of ten days, then on average 1% of the population will be off work on any given workday. An additional >1% would need to self-isolate due to being a close contact of an infected person. Consequently, high incidence makes it unlikely that quarantine orders can and will be adhered to or would even be mandated by some governments. Consequently, the effectiveness of this vital means to mitigate viral transmission is diminished.

Third, in the domain of education, infected children and their close contacts are excluded from attending school or childcare. In this manner, a high incidence also harms children and their education, even if schools remain open. This adds to the existing harms that children have already experienced during the pandemic.^23^

Fourth, a high incidence coupled with only part of the population being vaccinated or naturally immune after disease gives the virus more opportunities to mutate and increases evolutionary pressure on it to escape such immunity. This increases the probability of new VOCs emerging and spreading unnoticed in Europe.^24^ This is compounded by the fact that vaccinated people are unlikely to maintain the same vigilance levels regarding potential SARS-CoV-2 transmission as during the first phase of lockdowns.^25^ A VOC that renders existing vaccines less effective taking a foothold in Europe would prolong the pandemic and potentially cost even more lives and livelihoods.

Fifth, a central challenge is to avoid hospitals reaching and exceeding capacity. Assuming a slow COVID-19 incidence increase, largely effective vaccines, and fast progress in vaccination (including boosters where appropriate), hospitalization rates are unlikely to reach the levels of winter 2020/2021 (Figure 1). However, even if COVID-19-related hospitalizations remain substantially fewer than in previous waves, additional burdens will be placed on health systems: (a) With NPIs lifted and lowered risk perception, influenza, Respiratory Syncytial Virus, and pneumonia cases are likely to be more than last year^26^ and (b) due to postponement of surgeries and routine care during the pandemic there is a large backlog of patients in need of care.^27^ Indeed, if incidence increases before a sufficient proportion of people has been vaccinated (against COVID-19 and influenza), health systems may reach capacity limits (Figure 1) - with all this implies for quality of care and patient safety.

Finally, the economic, social, and health related burdens of NPIs should not be neglected either.^30^^,^^31^ Many of these burdens hit vulnerable and disadvantaged groups particularly hard. Maintaining and achieving low incidence is likely to reduce the need for the kinds of restrictions that are most harmful. Nevertheless, unintended negative consequences to ostensibly laudable measures are well characterised in the history of public health. As such, the role of NPIs in producing harm must be closely and carefully monitored.

In sum, strategies that seek to accommodate or accept high incidence at the current pandemic stage ironically lead to the very consequences they set out to prevent: With rising incidence, more invasive NPIs, potentially even lockdowns, become necessary. This, in turn, increases the negative effects both of the virus itself as well as the harms incurred by NPIs and other pandemic prevention and containment measures. Moreover, the zig-zag courses that pandemic measures inevitably take when too many NPIs are dropped too quickly make it more difficult to communicate to the public and for people and businesses to plan ahead. This adversely impacts upon the psychological wellbeing, whilst simultaneously eroding public trust.^28^

## A low incidence strategy to avoid illness, deaths, and lockdowns

An alternative to accepting a high COVID-19 incidence is to achieve and maintain a low incidence by combining increasing population immunization with moderate NPIs in the winter and progressive social and economic policy measures to enhance public health.^16^^,^^29^ The rationale for this recommendation rests on the following three pillars: First, when incidence is high, retaining control over viral transmission is much more difficult. At low incidence, in contrast, TTIS systems can function effectively.^14^^,^^15^ Second, as population vaccination coverage progresses - including younger age groups for whom vaccines are newly approved or authorised, the effective reproduction number R_eff_ is continuously reduced, necessitating only moderate NPIs to keep R_eff_ below 1. Finally, a key aim of low incidence is to avoid the more restrictive measures that would follow spikes in infection rates, consequently lessening the harms incurred by NPIs. For this reason, a strategy successfully maintaining a low incidence provides more stability and helps to protect from the manifold social, psychological, and economic harms of such more restrictive measures (see Table 1).

**Table 1:** Conditions and implications of corner scenarios for two hypothetical incidence regimes in Europe.

Overall, a pan-European commitment is crucial. The core pillars necessary to achieve and maintain low incidence include a clear political commitment across all Europe to rapidly achieve high vaccination coverage, the close and systematic monitoring of the spread of SARS-CoV-2 and its VOCs across regions and countries, and a systematic and representative sampling of SARS-CoV-2 infection among asymptomatic and symptomatic carriers together with monitoring of new variants presenting an early warning system. Should a new Delta wave occur in the winter, or should novel VOCs that can evade vaccines emerge, coordinated timing of NPIs across Europe will be essential to avoid the ‘ping-pong’ effects of cross-border spread.^29^ The better (and earlier) less-invasive NPIs such as the use of masks, the prohibition of mass gatherings, or good testing regimes are maintained, the lower is the risk that more invasive NPIs will be needed. Last but not least, a common European strategy is needed to share vaccines with countries that do not have sufficient supply. Coordinated global cooperation will greatly facilitate the pursuit of a low COVID-19 incidence strategy, and thus indirectly the suppression of the emergence of new VOCs. This would allow control of the pandemic and the discussed risks of the high-incidence scenario would be avoided.

In sum, at low incidence, further damage to health, economy and society could be prevented. Unlike in 2020, European countries now have the ability to effectively implement moderate NPIs (e.g., indoor facemasks, lateral-flow testing). We have a better understanding of the effectiveness of different NPIs than a year ago. This means that societies are, now, in a better position to choose the minimum and least invasive set of NPIs necessary to reach and maintain low incidence, alongside social and economic policy measures (such as easily accessible payments to enable self-isolation) that will likewise play a vital role in keeping cases low.

## Implementing a low incidence strategy - communication and trust

It remains critical that policies to mitigate and recover from the pandemic define a clear, timely, and accurate communication strategy early on, and include a sufficient degree of democratic involvement and coordination involving all stakeholders. Aside from the democratic imperative to do so, the effectiveness of rules and recommendations largely depends on the willingness and ability of populations to adhere to them.^31^ Opaque and ambiguous communication has been identified as an important reason for declining public trust and falling public commitment to pandemic measures.^1^^,^^3^ The importance of clear communication strategies that include scientific evidence and openly acknowledge uncertainties, are key to public trust.^32^

Faster progress should also be made on establishing mechanisms of actively tackling misinformation and systematically developing counter arguments regarding COVID-19 vaccines and NPIs.^33^ The support of locally respected persons, primary healthcare with strong links to communities, and the use of culturally adapted strategies can greatly contribute to sound messaging, with sincerity, openness, and empathy, to enhance public awareness and adherence.^34^^,^^35^ In addition, those residing in countries where measures are in place should have resources available to them for adherence to be economically and psychologically viable.^4^^,^^36^

## Conclusion

Despite important progress in vaccination coverage, and the ability to reach low incidence across many European countries over the summer months of 2021, there is a risk of a resurgence of COVID-19 cases in winter. This is especially important if vaccination rates among the most vulnerable groups of the population are insufficient. Decision makers should think ahead and take decisive measures to avoid the failures of 2020: Evidence-based proactive and effective regulations, instead of knee-jerk reactions, across Europe are needed, alongside bold and imaginative social and economic policy to support and enhance public health. With uncertainty regarding children and vulnerable groups, such as those immunocompromised, and, especially, with the catastrophically low availability of vaccines in the Global South, a high incidence in Europe will have global implications that will ultimately adversely impact everyone.

We have yet to overcome the pandemic, but an end is at least conceivable. Until then, the goal is to minimize the cost, emerging from the virus directly, and from measures to prevent its spread, to our communities and societies in Europe and across the world. The way to achieve this is with coordinated European commitment and cooperation, including a Pan-European voice within the global multilateral dialogue, to effectively control the transmission of SARS-CoV-2.

**Key Messages**

- While at this point of the pandemic incidence levels have a different meaning (due to vaccination), they remain very relevant for policymaking. For example, they correlate with the risk of long COVID, determine the effectiveness of test-trace-isolate-support programs, and predict the proportion of serious cases requiring hospitalisation.
- A high incidence strategy may seem the easiest route for political decision makers, but it is fraught with risks, provides less stability, and ultimately incurs higher costs.
- A low-incidence strategy in Europe seems achievable and more advantageous for public health, society, and for the economy.
- To achieve low incidence, a moderate and targeted set of NPIs should be maintained or re-introduced alongside progressive social and economic policy that enables social practices aligned with the goal of decreasing the impacts of the pandemic until vaccination coverage is sufficient.
- Pan-European commitment and cooperation is key to the success of this strategy.

Alt-text: Unlabelled box

## Author contributions

Most authors were part of a Delphi study on the future of the Covid-19 pandemic and a follow-up opinion piece.^4^^,^^35^ The idea for this article emerged from these discussions. TC, ENI, BP, VP conceived the article, wrote the first draft, and are first authors. All authors provided expertise from their respective fields and were involved in writing, review, and editing. TC, ENI, and BP coordinated the shaping of the paper.

## Declaration of Competing Interest

TC was supported by the EU Commission, grant agreement No 101016233 (PERISCOPE). SB was supported by Netzwerk Universitätsmedizin, project egePan (01KX2021). ACV's institution was supported by Ministry of Culture and Science of the German State of North Rhine-Westphalia. EGl was supported by the Luxembourg National Research Fund (FNR) with Public funding support with payments to the host institute as part of the COVID-19 Fast-Track grant research project CovScreen (COVID-19/20201/14715687). EGr has received payments for a manuscript on the history of pandemics. JK is employed by a project funded by the European Research Council, European Union's Horizon 2020 research and innovation programme (grant agreement no. 724460). CL received grants from the University of Oxford, National Centre for Smoking Cessation and Training, UK, Horizon 2020, EUROPEAN COMMISSION, and Pfizer Inc, royalties from Olvos Science, payment for expert testimony from Word Health Organization and European Commission, has a patent for Cretan Iama Olvos Science, and is on the advisory board for Pfizer Helas and Vianex SA. GNP received grants and royalties from Novartis, FNIH, Gilead Grants, managed through NIH, and is the chair of the Nemitsas Prize Award Committee. MPi was supported by Wellcome Trust [grant numbers: 209519/Z/17/Z; WT106612MA], MRC [grant number: MR/S035818/1], ESRC [grant numbers: ES/T014164/1; ES/S013873/1], and British Academy [EN160164]. ESz's lab receives funding for other projects from Merck Healthcare. ST's institution received grants due to his role as Co-investigator-PI in study under the European Union's Horizon 2020 research and innovation programme, Grant Agreement, No 883441, under the agreement and control of the Special Committee for Research Grants of the University of Athens, Athens, Greece. PWilmes’ institution received grants from the European Commission's Horizon 2020 programme including the European Research Council (CoG 863664), the Luxembourg National Research Fund, and the University of Luxembourg, and owns patents. PWilmes received honoraria for being on two PhD juries at the University of Copenhagen and for the Maud Menten lecture at the University of Western Ontario, and for membership of the scientific steering committee for a clinical trial by 4D Pharma plc. and he is Co-speaker of the Research Luxembourg COVID-19 Task Force. Vice-president of the Luxembourg Society for Microbiology. All these were unrelated to this article. All other authors declare no competing interests.
